# On the stratospheric chemistry of midlatitude wildfire smoke

**DOI:** 10.1073/pnas.2117325119

**Published:** 2022-03-01

**Authors:** Susan Solomon, Kimberlee Dube, Kane Stone, Pengfei Yu, Doug Kinnison, Owen B. Toon, Susan E. Strahan, Karen H. Rosenlof, Robert Portmann, Sean Davis, William Randel, Peter Bernath, Chris Boone, Charles G. Bardeen, Adam Bourassa, Daniel Zawada, Doug Degenstein

**Affiliations:** ^a^Department of Earth, Atmospheric, and Planetary Sciences, Massachusetts Institute of Technology, Cambridge, MA 02139;; ^b^Institute for Space and Atmospheric Science, University of Saskatchewan, Saskatoon, SK, S7N 5A2 Canada;; ^c^Institute for Environmental and Climate Research, Jinan University, Guangzhou 510630, China;; ^d^Atmospheric Chemistry Observations and Modeling, National Center for Atmospheric Research, Boulder, CO 80307;; ^e^Department of Atmospheric and Oceanic Sciences, Laboratory for Atmospheric and Space Physics, University of Colorado, Boulder, CO 80303;; ^f^Goddard Earth Science Technology and Research (GESTAR), NASA Goddard Space Flight Center, Greenbelt, MD 20771;; ^g^NOAA Chemical Sciences Laboratory, Boulder, CO 80305;; ^h^Department of Chemistry and Biochemistry, Old Dominion University, Norfolk, VA 23529;; ^i^Department of Chemistry, University of Waterloo, Waterloo, ON, N2L 3G1 Canada

**Keywords:** stratospheric ozone, wildfire, chemistry, smoke

## Abstract

Massive Australian wildfires lofted smoke directly into the stratosphere in the austral summer of 2019/20. The smoke led to increases in optical extinction throughout the midlatitudes of the southern hemisphere that rivalled substantial volcanic perturbations. Previous studies have assumed that the smoke became coated with sulfuric acid and water and would deplete the ozone layer through heterogeneous chemistry on those surfaces, as is routinely observed following volcanic enhancements of the stratospheric sulfate layer. Here, observations of extinction and reactive nitrogen species from multiple independent satellites that sampled the smoke region are compared to one another and to model calculations. The data display a strong decrease in reactive nitrogen concentrations with increased aerosol extinction in the stratosphere, which is a known fingerprint for key heterogeneous chemistry on sulfate/H_2_O particles (specifically the hydrolysis of N_2_O_5_ to form HNO_3_). This chemical shift affects not only reactive nitrogen but also chlorine and reactive hydrogen species and is expected to cause midlatitude ozone layer depletion. Comparison of the model ozone to observations suggests that N_2_O_5_ hydrolysis contributed to reduced ozone, but additional chemical and/or dynamical processes are also important. These findings suggest that if wildfire smoke injection into the stratosphere increases sufficiently in frequency and magnitude as the world warms due to climate change, ozone recovery under the Montreal Protocol could be impeded, at least sporadically. Modeled austral midlatitude total ozone loss was about 1% in March 2020, which is significant compared to expected ozone recovery of about 1% per decade.

## Significance

Large wildfires have been observed to inject smoke into the stratosphere, raising questions about their potential to affect the stratospheric ozone layer that protects life on Earth from biologically damaging ultraviolet radiation. Multiple observations of aerosol and NO_2_ concentrations from three independent satellite instruments are used here together with model calculations to identify decreases in stratospheric NO_2_ concentrations following major Australian 2019 through 2020 wildfires. The data confirm that important chemistry did occur on the smoke particle surfaces. The observed behavior in NO_2_ with increasing particle concentrations is a marker for surface chemistry that contributes to midlatitude ozone depletion. The results indicate that increasing wildfire activity in a warming world may slow the recovery of the ozone layer.

Recent large-scale wildfire events in many parts of the world including British Columbia in 2017 (1) and the Australian “black summer” fires in 2020 (2, 3) have injected substantial loadings of smoke particles directly into the stratosphere via the outflow from towering Pyrocumulonimbus (PyroCb) towers. While wildfires have occurred for many thousands of years, evidence suggests that their scale and frequency are increasing with global warming (4). The properties and composition of wildfire smoke particles have been studied in the troposphere and stratosphere using both in situ and lidar methods (e.g., refs. 5–8), and stratospheric smoke plume heights have been documented with satellite observations (9, 10). Further, repeated transects through fire smoke observed by instruments onboard passenger aircraft have shown that wildfire smoke particles acquire a liquid coating (11), a finding supported by multiwavelength lidar studies (5, 12). Satellite observations revealed large increases in organic species in the gas phase associated with stratospheric smoke (13), including acetone and CH_3_OH (as would be expected due to incomplete combustion in biomass burning). They also provided spectral evidence that the smoke particles themselves contained organic material (e.g., carboxylic acids as noted in wood smoke in ref. 14). Stratospheric, single-particle measurements of smoke have detected internally mixed particles containing not only organic compounds (i.e., black and organic carbon) but also sulfates (8).

Stratospheric aerosol extinction ratios (relative to a purely molecular atmosphere) associated with the 2020 Australian fires were comparable to those following the eruption of the Calbuco volcano in April 2015 and blanketed the Southern Hemisphere (SH) midlatitudes (3, 15). Even under nonvolcanic conditions, the stratosphere contains a layer of liquid sulfuric acid/water particles that can drive significant midlatitude ozone depletion chemistry (16). Sufficiently explosive volcanic eruptions inject additional sulfur into the stratosphere, which ultimately increases the sulfuric acid abundances and can greatly enhance the particle surface areas. Reactions occurring on such particles affect reactive nitrogen (NO and NO_2_; the sum of the two is called NO_x_ here). NO and NO_2_ exchange rapidly with one another in the daytime stratosphere depending on ozone abundances, temperatures, and photolysis rates. Therefore, the sum of both species is more robust to variability in temperature, ozone, or solar angle than either alone. NO_x_ reductions in turn affect ClO and OH radicals, and all of these species participate in catalytic cycles that deplete ozone. Major volcanic eruptions of the past half-century have been shown to enhance midlatitude stratospheric ozone destruction (17–20). The 2015 Calbuco event resulted in observable reductions in midlatitude SH ozone concentrations in the lower stratosphere, consistent with calculations of chemical depletion (21). The 2020 Australian fires were associated with similar SH ozone reductions (15). The smoke’s radiative properties also locally warmed the lower stratosphere by up to a few degrees (15, 22).

A detailed model study of the 2020 Australian wildfire particles assumed that the particles became coated with sulfuric acid (22) and hence displayed similar midlatitude chemistry to background and volcanic stratospheric aerosols (*Materials and Methods*). That work estimated that heterogeneous reactions involving wildfire-enhanced aerosols could reduce SH midlatitude stratospheric ozone by about 5 to 10 Dobson Units (DU) from July to August of 2020. However, some studies have argued that wildfire smoke might form glassy surfaces (6) in the lowermost stratosphere, which would likely display quite different chemical reactivity from liquids. Tropospheric studies have shown that wildfire particles contain differing mixes of soot, primary organics, and secondary organic compounds as well as minerals and salts (23), dependent on such factors as the type of fuel (rainforest, woodland, etc.) and state of the fire (smoldering, flaming, etc.). While some studies have suggested somewhat-reduced uptake of N_2_O_5_ when aerosols are coated with organics (e.g., refs. 24, 25), other work indicates differing behavior depending upon specific composition and such factors as whether the organic coatings are straight chain or branched (e.g., ref. 26). Therefore, observations and modeling studies that can improve the understanding of the impacts of wildfire smoke on stratospheric composition and chemistry are needed and are the goal of this paper.

We use satellite observations of NO_x_ species and aerosols together with model simulations (from ref. 22) to examine the role of the 2020 Australian wildfire smoke in midlatitude stratospheric NO_x_ chemistry. The abundance of stratospheric NO_x_ has long been known to be a key marker for midlatitude heterogeneous chemistry on liquid sulfate aerosols, particularly when aerosols are enhanced (e.g., in major volcanic eruptions) (17, 27) as described in *Results*. We demonstrate that the satellite NO_x_ observations provide strong evidence that stratospheric wildfire smoke drives important chemistry that can be expected to contribute to ozone depletion as long as stratospheric chlorine abundances remain elevated.

## Results

We make use of three satellite records to examine the behavior of stratospheric reactive nitrogen after the Australian fires [i.e., NO_2_ data from the Optical Spectrograph and InfraRed Imager System {OSIRIS} (28), the Stratospheric Aerosol and Gases Experiment on the International Space Station {SAGEIII/ISS} (29), and the Atmospheric Chemistry Experiment {ACE} (30)]. We also present ∼750-nm extinction ratio data from both OSIRIS and SAGE III as well as from the Ozone Mapping and Profiler Suite Limb Profiler (OMPS/LP) from (31, 32). SAGE III/ISS and OSIRIS both employ absorption for NO_2_ measurement at visible wavelengths, while ACE uses Fourier transform infrared spectroscopy. All three make use of limb-viewing geometry, either through direct solar occultation (SAGE III/ISS and ACE) or limb scattering (OSIRIS).

**Fig. 1.**
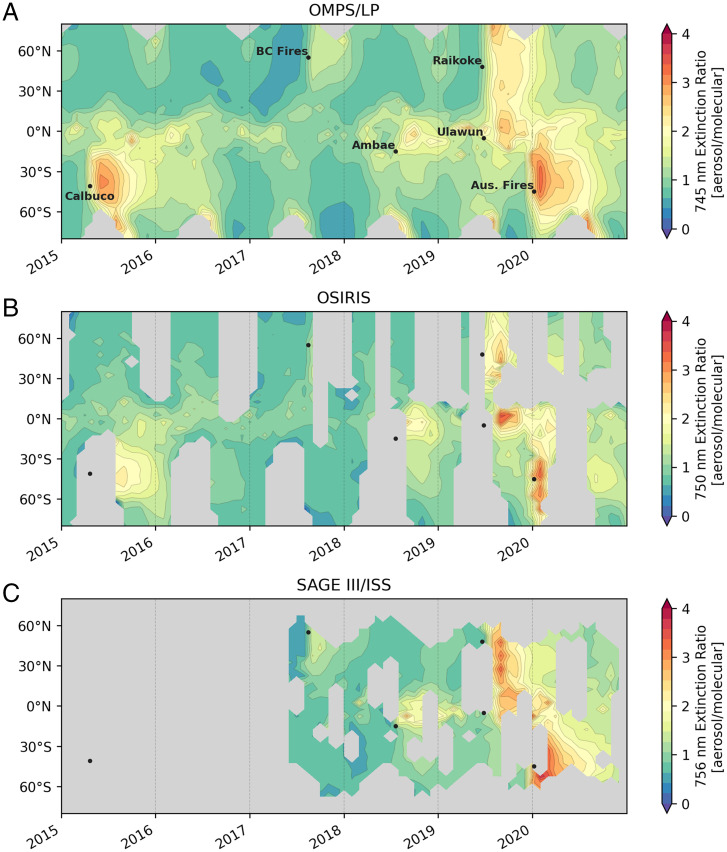
Monthly mean lower stratospheric aerosol extinction observations at around 750 nm (defined as the ratio to gas phase molecules only), available since 2015 from OMPS (*A*), OSIRIS (*B*), and SAGE III/ISS (*C*). Data represent an average for the lower stratosphere, weighted by the microwave limb sounder temperature weighting function, which is centered around 100 mb or about 16-km altitude (50). The year 2015 is selected as the start date to capture the Calbuco eruption, which displayed similar SH extinction levels to those obtained after the 2020 Australian fires. Gray regions indicate missing values (*Materials and Methods*). SAGE III/ISS and OSIRIS data shown here are the average of sunrise and sunset occultations.

Fig. 1 presents monthly averaged stratospheric aerosol extinction ratio time series from the three instruments, demonstrating broad consistency between the datasets. The observations display a large perturbation to SH midlatitude aerosol extinction ratio due to the 2020 Australian fires. As noted in previous studies using OMPS (3), the midlatitude aerosol extinction ratio perturbation after these fires was comparable to that following the substantial eruption of Mount Calbuco in 2015; the OSIRIS and SAGE data shown in Fig. 1 provide independent support for this conclusion. The eruption of the Ulawun volcano in the tropics in 2019 affected the aerosol loading in the tropics shortly before the 2020 fires in all three datasets. While coverage in OSIRIS and SAGE III/ISS is more limited due to their limb-viewing geometries, the three instruments nonetheless suggest similar timing and spread of the 2020 wildfire smoke. *SI Appendix*, Fig. S1 presents the extinction ratios at 675 nm as estimated by the model in ref. 22 and shows good general agreement with OMPS despite the small difference in the wavelengths available for each. Smoke particles accumulate water, increasing extinction and providing added surface area to drive faster heterogeneous chemistry, just as added sulfate does following volcanic eruptions, but with different hygroscopicity (*Materials and Methods*).

**Fig. 2.**
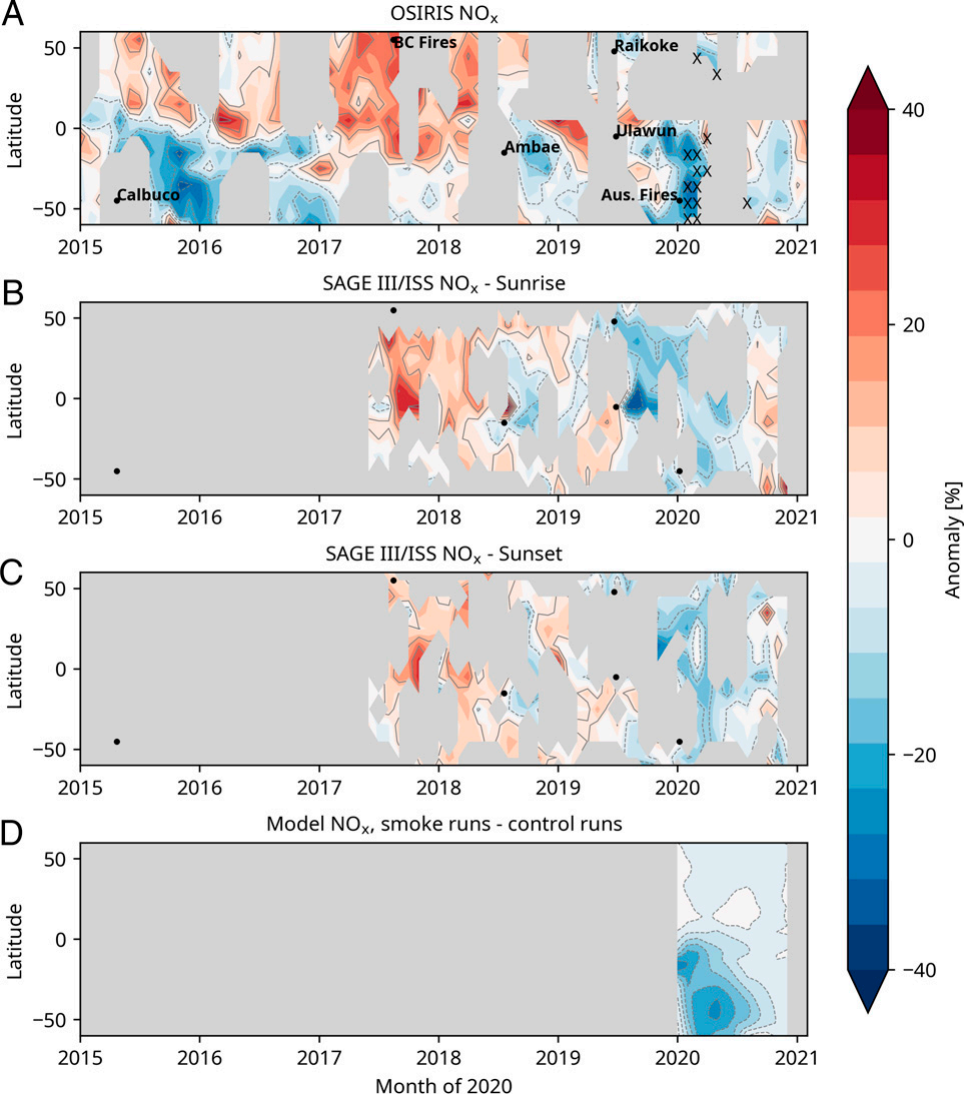
Monthly averaged 18.5-km level anomalies (percent) in deseasonalized available years of OSIRIS NO_x_ (*A*) and sunrise (*B*) and sunset (*C*) SAGE III/ISS data along with the difference between the smoke and no-smoke model runs for 2020 (*D*). Gray regions in the data indicate missing values (*Materials and Methods*). The hatched regions on the OSIRIS panel show where the 2020 anomaly is greater than the maximum or less than the minimum anomaly over all the data from 2002 to 2019.

Fig. 2 displays the corresponding monthly averaged satellite NO_x_ anomalies obtained from the OSIRIS and SAGE III/ISS NO_2_ data at 18.5 km (*Materials and Methods*) along with the NO_x_ change calculated in the model. OSIRIS data indicate that 2020 NO_x_ was lower than all previous years since 2002 throughout a broad range of latitude for multiple months, from 30 to 60°S (Fig. 2), so this region was selected for focused study in this paper. OSIRIS data display larger variability at lower latitudes (particularly below about 19 km), making identification of wildfire impacts challenging equatorward of about 30°S. Further, Ulawun may have perturbed tropical NO_x_, but the abrupt NO_x_ change in early 2020 (at least for latitudes poleward of 30°S) suggests that the fires dominated at these latitudes. SAGE III/ISS NO_x_ data show very similar timing and spread of the midlatitude anomaly to OSIRIS, albeit with more-limited coverage. ACE NO_2_ data also have limited coverage and are shown in *SI Appendix*, Fig. S2; these are not converted to NO_x_ here both because of coverage limitations and because ACE measures both NO and NO_2_ directly, but the NO retrieval is still under development. Nonetheless, ACE NO_2_ data display consistent features to the other datasets. Observed 2020 anomalies in reactive nitrogen species at 18.5 km from 30 to 60°S relative to other available years are at least 20% or larger in all three satellite datasets, a large change. Magnitudes of the NO_x_ perturbations from OSIRIS and SAGE data are different in part because of differences in coverage as well as the number of available sunrise versus sunset data points in each. Fig. 2 also shows the spread of the ensemble mean NO_x_ change at 18.5 km calculated in the model, defined as the difference between the smoke versus no-smoke runs, and the model is in good general agreement with the data. Note that the model calculations shown here did not include the Ulawun eruption and thus reflect purely the calculated NO_x_ change from smoke particle chemistry. A latitude height plot for March comparing the model and OSIRIS data are presented in *SI Appendix*, Fig. S3, again showing broad consistency between the model and the data. The 18.5-km altitude was selected for focus in this paper in order to balance OSIRIS data quality (better at higher rather than lower altitudes) and levels displaying extensive SH NO_x_ perturbations as shown in Fig. 2 and *SI Appendix*, Fig. S3.

**Fig. 3.**
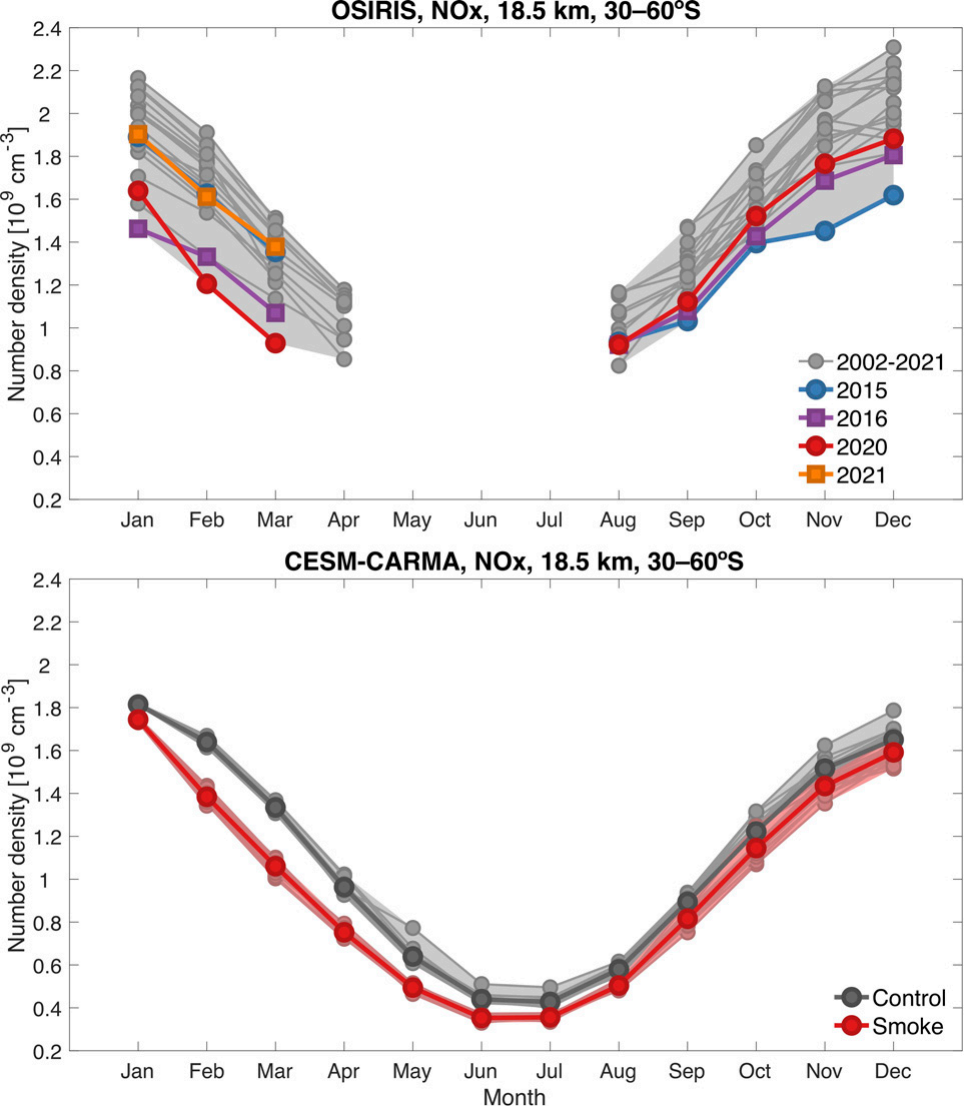
NO_x_ concentrations (molecules/centimeter^3^) by month, averaged from 30 to 60°S at 18.5 km. The *Top* panel presents 20 y of OSIRIS measurements, with 2015 and 2016 (after the Calbuco eruption) and 2020 and 2021 (after the Australian black summer fires) distinguished from others by the indicated colors. The *Bottom* panel shows 20 realizations of 2020 both with (red) and without (gray) smoke for the same latitudes and altitude as calculated in the model.

While dynamical contributions to the anomalies cannot be ruled out, Fig. 3, *Top* probes this region in more detail and shows that for February and March monthly averages at 18.5 km, the OSIRIS NO_x_ amounts are lower than observed in any previous year of the available 20-y record, strongly suggestive that the wildfires drove the change. ACE data presented in *SI Appendix*, Fig. S4 also display record lows for March and April 2020 in a record spanning 17 y. The chemical mechanism responsible is discussed further below. The OSIRIS data also indicate a large influence of the Calbuco aerosols on NO_x_, beginning in the latter half of 2015 and extending into 2016. Indeed, while 2020 OSIRIS data show the lowest NO_x_ observed in this region in February and March, the second lowest is 2016 after Calbuco. By August 2020, the wildfire smoke impact on NO_x_ appears to have diminished in the OSIRIS observations, and concentrations in the latter half of the year are within the range of other years. Fig. 3, *Bottom* presents the calculated NO_x_ concentrations from 20 realizations in the model for the smoke and no-smoke cases. The OSIRIS observations for March indicate about 1 × 10^9^ molec/cm^3^ after the fires versus about 1.4 × 10^9^ in other years, and the model results are very close to these values.

The primary chemical mechanism driving NO_x_ reductions with increasing stratospheric sulfate aerosols and its implications for midlatitude ozone losses have long been known (16, 17, 33). Even for background aerosols, these processes decrease midlatitude ozone column abundances by several percent compared with estimates using only gas-phase chemistry for current levels of stratospheric chlorine loading. As chlorine abundances diminish in the future because of the phaseout of chlorofluorocarbons under the Montreal Protocol, the ozone depletion can be expected to decrease and eventually flip sign to positive values (34), but depletion can be expected through the mid-21st century. Further, these reactions were responsible for enhanced midlatitude ozone destruction following several past volcanic eruptions (e.g., El Chichon and Pinatubo) (19, 20).

The principal lower stratospheric photochemical mechanism is well established: NO is converted entirely to NO_2_ at night, which goes on to form NO_3_ and then to N_2_O_5_. The NO_3_ intermediate photolyzes rapidly in daytime, so the formation of N_2_O_5_ is only rapid at night. Hence, N_2_O_5_ is an important nighttime reservoir for NO_x_. A critical reaction under warm midlatitude conditions is the heterogeneous hydrolysis of N_2_O_5_, which converts reactive nitrogen to HNO_3_—a process that does not occur in the gas phase. N_2_O_5_ photolyzes fairly rapidly during the day (order of hours), while HNO_3_ photolysis is much slower in the lower stratosphere (order of a week or more). Nighttime conversion of N_2_O_5_ to HNO_3_, therefore, reduces NO_x_, which in turn means that the NO_2_ concentration available to form ClONO_2_ is reduced, leading to an increase in ozone-destroying ClO. Reductions in NO_x_ influence HO_x_ radicals as well (19, 35), which are also important for ozone-loss chemistry. N_2_O_5_ hydrolysis on sulfuric acid/water particles has been extensively studied in the laboratory and occurs with high efficiency (36) at essentially all atmospheric temperatures. More-recent studies have shown that BrONO_2_ hydrolysis is also important for heterogeneous HNO_3_ formation under these conditions (37), while ClONO_2_ hydrolysis contributes at colder conditions (i.e., temperatures below about 195K) (33). Here, we use satellite observations to probe whether similar composition changes occur due to wildfire smoke. Because HNO_3_ concentrations are much larger than those of NO_x_ at the altitude range considered, NO_x_ is a better indicator of this chemistry than HNO_3_ would be. Observations also indicate that some HNO_3_ was taken up by these particles (38), perhaps due to their high organic content (39).

A key point first made by ref. 17 is the role of nonlinear chemistry that occurs with increasing aerosol loading. While the rate of N_2_O_5_ hydrolysis increases rapidly at lower aerosol content, the reaction saturates when HNO_3_ is formed fast enough to remove essentially all the N_2_O_5_ formed in a given night, due to slow release by HNO_3_ photolysis the following day in the lower stratosphere. Further increases in aerosols, then, cannot significantly increase the reaction rate, because N_2_O_5_ is already being destroyed as fast as it can be produced (i.e., formation of NO_3_ and hence N_2_O_5_ through the nighttime NO_2_+O_3_ reaction becomes the rate-limiting step).

**Fig. 4.**
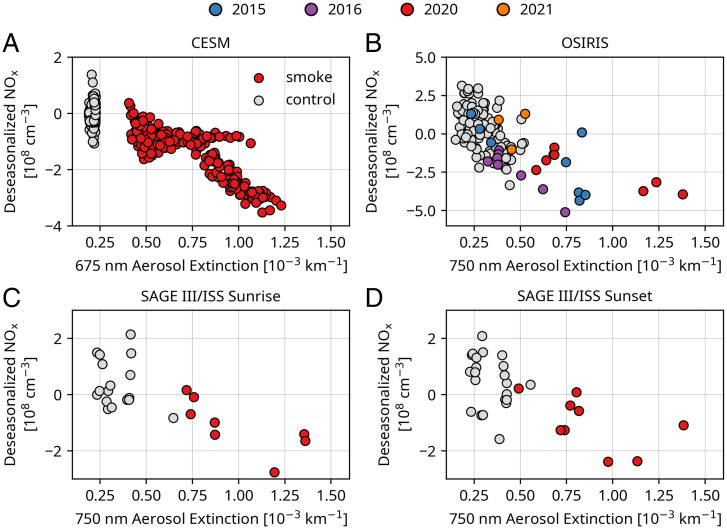
Monthly mean deseasonalized NO_x_ versus aerosol extinction at 18.5 km and from 45°S to 40°S for the model (*A*), OSIRIS (*B*), and SAGE III/ISS (*C* and *D*). The years 2015 and 2016 (after the Calbuco eruption) and 2020 and 2021 (after the Australian black summer fires) are in colors, while the other years make up the gray points. Outliers greater than four median absolute deviations from the median were removed from the OSIRIS NO_x_ and SAGE III/ISS NO_x_ data. Model points are averaged results for each month from each of the 20 ensemble members.

This heterogenous chemistry leads to a characteristic curve of decreasing NO_x_ abundances versus increasing aerosols (17), a diagnostic fingerprint of this chemistry. Fig. 4 presents such curves for 40 to 45°S at 18.5 km using available SAGE III/ISS sunrise and sunset NO_x_ data, OSIRIS NO_x_ data, and NO_x_ calculated in the smoke model. Observations and model results are deseasonalized by month using all available years of data for each instrument (*Materials and Methods*). High extinction values are observed without low NO_x_ in January 2020 when the plume had freshly entered the stratosphere, suggesting that the timescale for the chemistry is of order 1 mo. The 2020 observations reveal the expected decay in agreement with the model. We note that the rate-limiting gas-phase reaction NO_2_+O_3_ → NO_3_ + O_2_ is faster by about 7% due to the smoke-induced warming in the March ensemble mean (212.15 K versus 210.9 K) at 40 to 45°S and 18.5 km. This temperature change is, however, only a small contribution to the modeled NO_x_ changes compared to the more-than-threefold March surface area change and hence increased N_2_O_5_ hydrolysis rate due to the smoke. The OSIRIS data suggest that the peak 2020 NO_x_ reduction driven by the wildfires may have exceeded that from the Calbuco volcano, but it should be recalled that OSIRIS did not sample this region immediately after that eruption and did promptly sample the smoke. OSIRIS data suggest that the N_2_O_5_ hydrolysis reaction reached its saturation limit on the wildfire particles, a finding also suggested by the SAGEIII/ISS data albeit less clearly because of the limited coverage. The model is less clear regarding saturation but is in broad agreement with the decline. *SI Appendix*, Fig. S5 shows that similar behavior is observed in the ACE NO_2_ record as well, despite limitations of coverage and differences in the wavelengths of the extinction measurements.

Fig. 4 strongly supports the view that the Australian wildfire particles drive hydrolysis of N_2_O_5_ in a manner that is similar to sulfate particles. Thus, the presence of organic matter along with sulfate (15) apparently did not render the particles sufficiently glassy to inhibit the uptake of water needed to allow N_2_O_5_ hydrolysis. Fig. 4 supports the view taken by Yu et al. (22) that the 2020 wildfire aerosols behaved like sulfate particles insofar as their midlatitude heterogeneous chemistry is concerned.

We next compare modeled and observed midlatitude ozone changes but do not consider Antarctic ozone hole behavior. Polar stratospheric clouds (PSCs) are responsible for the extreme austral springtime ozone losses found in the Antarctic through heterogeneous chlorine and bromine chemistry (40, 41) and are enhanced after volcanic eruptions (42). They are composed in part of liquid sulfuric acid, water, and nitric acid. It is plausible that the Australian smoke particles may have enhanced PSC reactions and perhaps influenced midlatitude ozone indirectly through transport of reduced ozone values from the ozone hole, but this chemistry is not examined here. The model used here did not include the wildfire aerosols in their PSC reaction set, allowing us to isolate the midlatitude chemistry alone (i.e., as distinct from any transport from the ozone hole region at polar latitudes) with high confidence.

**Fig. 5.**
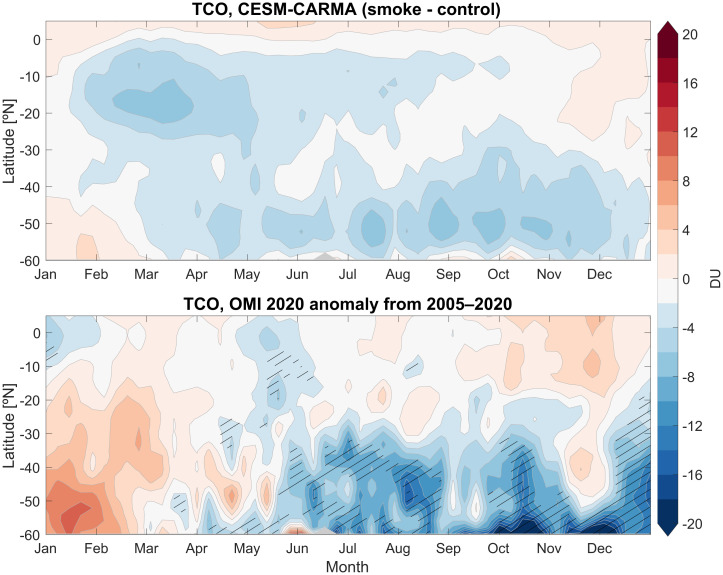
Calculated weekly averaged changes in total column ozone for the ensemble mean of the smoke minus no-smoke runs in the model (*Top*). These model runs did not allow smoke particles to pick up HNO_3_ and form PSCs. Observed anomalies in 2020 total column ozone from OMI observations (*Bottom*). The OMI data represent anomalies calculated after the time series has been linearly detrended over the period from 2005 to 2020 (*Materials and Methods*). Large negative anomalies during 2020 are indicated by line-hatching if they are lower than the 25th percentile.

Fig. 5 compares weekly and zonally averaged observed total ozone anomalies to the changes obtained between the smoke versus no-smoke model runs (*Materials and Methods*). The differences between smoke and no-smoke runs explicitly isolates the impact of the chemistry included in the model, while the observational anomalies will reflect not only these chemical effects but any others that may be occurring, as well as any dynamical changes. The total ozone anomalies at southern midlatitudes from the free-running ensemble mean of the model results display important similarities in morphology with time and latitude but are considerably smaller than observed. Ozone reductions at low latitudes near −10 to −20°S in the model are not observed in the data, perhaps due to dynamical variability or incomplete smoke chemistry. Low ozone anomalies (lowest 25th percentile of the record) occur near −50 to −55°S in late March but are larger than that suggested in the model, and variable increases also seen near −40 to −55°S in April to May suggest dynamical fluctuations. Notably, Ozone Monitoring Instrument (OMI) observes low total ozone in the −40 to −50°S latitude band from late May through August 1, too early in the year for substantial polar depletion (although transport from polar regions may well contribute later in the year). The model also suggests reduced ozone from smoke chemistry throughout this period at those latitudes, but the calculated ozone loss is smaller than observed. Dynamical variability could contribute to the observed changes, and it is plausible that the heating from the smoke plume altered the stratospheric circulation; these factors are not examined here. A combination of dynamical variability and chemical contributions to the anomalous midlatitude ozone changes may be occurring, or additional chemical processes on the smoke particles not considered here may be important. Overall, the comparison suggests that the smoke chemistry indicated by the NO_x_ observations as represented in this model did contribute to ozone reductions that appear to occur in the observations but also shows that other factors are highly likely to be important.

## Discussion

Multiple satellite datasets for stratospheric aerosol extinction and NO_x_ perturbations following the Australian wildfires of 2020 have been compared to one another and to recently published model calculations in this paper. Record-low NOx abundances in the SH midlatitude lower stratosphere were measured by OSIRIS and ACE. SAGE III/ISS observations only extend over about the past 3 y but also display exceptionally low NO_x_ in 2020, comparable to the anomalies found in the other two instruments. The observed seasonal and latitudinal changes in NO_x_ near 18.5 km are broadly consistent with modeling results. Most importantly, the satellite data from both OSIRIS and SAGE III/ISS indicate large decreases in NO_x_ abundances, which saturate with increasing aerosol extinction values, in good agreement with the model. This characteristic behavior has been previously studied (17). Its occurrence in three sets of satellite measurements presented here strongly suggests that the wildfire aerosols drove hydrolysis of N_2_O_5_ on wet particles. Thus, the evidence indicates that this key heterogeneous reaction was indeed enhanced on the Australian fire smoke surfaces, just as it is following major volcanic eruptions including (e.g., Calbuco in the SH in 2015). The impact of that volcanic eruption on NO_x_ was also shown to be discernible in the OSIRIS data but was smaller than that obtained following the 2020 fires in the monthly averages for February and March.

Decreased NO_x_ and NO_2_ related to N_2_O_5_ hydrolysis is expected to be accompanied by increases in HO_x_ and ClO, which drive net decreases in midlatitude ozone following major volcanic eruptions (19, 20). Decreases in midlatitude SH ozone of up to 8 DU were also observed after the Australian fires, and these reductions began prior to the formation of the springtime Antarctic ozone hole, indicating a local origin rather than transport of low ozone air from the ozone hole. Model results display chemical ozone decreases from about −40 to −50°S that are similar in their evolution from March through August but smaller in magnitude (with peak values of about 15 DU). The discrepancy may be due to dynamics or to added chemistry not represented in the model. We note that the Australian smoke was unusual in that it came largely from eucalyptus trees (3, 6), and whether similar chemistry can occur on other sources of stratospheric smoke more typical of other landscapes is not known. Laboratory work to elucidate the heterogeneous reaction rates that may occur in the stratosphere on mixed organic/sulfate particles is badly needed as well as field and ongoing satellite observations to better understand their composition and chemistry.

Overall, this work provides strong evidence that the Australian forest fires of 2020 resulted in chemical impacts on midlatitude stratospheric NO_x_ in a manner similar to that observed following volcanic eruptions. Modeled austral midlatitude total ozone loss was about 1% in March 2020, which is significant in magnitude (albeit limited in space and time) as compared to expected ongoing SH midlatitude ozone recovery due to the Montreal Protocol of about 1% per decade (43). The results suggest that this chemistry contributed to but did not fully capture the observed ozone changes following the fires. These findings are important given the uncertainties surrounding the chemistry that may occur on and in smoke particles. Our findings are suggestive that the Australian fire smoke did behave like sulfate aerosols and might therefore also have affected liquid PSCs and the Antarctic ozone loss in 2020 as well. Further work is required to examine that important chemistry and dynamics in detail. Finally, evidence strongly suggests that wildfire frequency and spatial extent has already increased and will continue to increase in the future due to climate change until ecosystem changes reduce available fuels. Our findings support the view that heterogeneous chemistry on wildfire smoke particles from PyroCb that reach the stratosphere represents an important chemistry–climate coupling mechanism that temporarily decreased SH midlatitude ozone in 2020. Future fires in a warming world could display larger or more-persistent impacts if wildfires become more frequent and/or intense, but there are many uncertainties including the chemical unknowns discussed herein. This paper has highlighted the need for further examination both of the chemistry of wildfire smoke in the stratosphere and the projected recovery of the ozone layer using coupled chemistry–climate–vegetation models.

## Materials and Methods

NO_2_ data are available since 2002 from the OSIRIS instrument based upon limb-scattered solar radiation, version 7.1. OSIRIS data have previously been compared to other measurements including the solar occultation method for NO_2_ employed by SAGE III/ISS version 5.1 (29). Broad agreement between the SAGE III/ISS dataset and OSIRIS NO_2_ has been demonstrated (44). The OSIRIS data for the morning orbit node are shifted to a common local solar time of 12:00 PM. OSIRIS and SAGE III/ISS data are converted to NO_x_ using the photochemical box model described in ref. 45. NO_2_ observations are also presented in the supplement from the ACE, which employs Fourier transform infrared spectroscopy in solar occultation mode at sunrise and sunset as well (46). Total ozone data are from the OMI (47).

Monthly mean values are calculated for each of the instrument datasets for any month containing at least five measurements. High-latitude winter data are always missing in OSIRIS, SAGEIII/ISS, and ACE due to lack of sunlight for the measurement. SAGE III/ISS and ACE data are generally limited by their orbits and occultation opportunities. Other gaps indicate data dropouts, especially for the aging OSIRIS instrument. The data are deseasonalized by subtracting the overall mean value for a given month of the year from that month (i.e., the overall mean January is subtracted from each individual January).

Extinction ratio data at ∼750 nm are available from both OSIRIS and SAGE III as well as a third instrument, the OMPS/LP. ACE includes two imagers, which measure aerosol extinction at 525 nm and 1,020 nm. OMPS extinction data shown here use the tomographic retrieval developed at the University of Saskatchewan (32). SAGE III/ISS and OSIRIS both measure limb extinction at visible/near infrared wavelengths, providing a useful comparison to the tomographic inversion approach used with OMPS.

Observations are compared to modeling results for the 2019 through 2020 Australian fires from the Community Aerosol and Radiation Model for Atmospheres (CARMA) coupled with the Community Earth System Model (CESM-CARMA) presented in ref. 22. The model includes 56 vertical layers from the surface of the Earth to about 45 km and a resolution of about 2° in latitude and longitude. The model was spun up in specified dynamics mode nudged to the Goddard Earth Observing System version 5 analysis from midsummer to the end of 2019, after which 20 perturbed initial condition runs were carried out in free-running mode from December 29, 2019, to the end of 2020. Smoke was input from December 29 to 31, 2019, and on January 4, 2020, the dates when PyroCb were observed in the stratosphere (48).

The 20 smoke ensemble members are paired with a control run (no-smoke) with the same initial conditions. Several test cases were conducted to probe the sensitivity of the aerosol abundances and lofting to the amount of injected material and the percentage of black carbon in the initial smoke plume. Results are shown from the case that agreed best with observations, in which it was assumed that the amount of smoke was three times that injected by the Pacific Northwest wildfire events and 2.5% black carbon.

Heterogeneous chemistry and extinction are enhanced in hygroscopic particles that pick up water and swell, providing increased surface areas as well as water content. Like sulfate aerosols, organics are assumed to pick up water in the model but with a lower hygroscopicity. The adopted hygroscopicity of sulfate is 0.8, while the adopted hygroscopicity of organics is 0.5 and that of black carbon is 0.1. Therefore, the smoke particle sizes are not as large as those that would occur for comparable sulfate particles. We determine the swelling of the mixed particles differently from pure sulfuric acid, based on weight percent calculation. Details are in ref. 49 (A6.1 for pure sulfuric acid and A6.2 for mixed particles). Available model calculations of extinction ratio used here are at a slightly different wavelength than the observations, 675 nm.

## Supplementary Material

Supplementary File

## Data Availability

All study data are included in the article and/or *SI Appendix*. Previously published data were used for this work (Model output used is available at https://osf.io/6j8cb/?view_only=72f53447bf464a2bbcc1dfc32d492bab. OSIRIS data are available at https://research-groups.usask.ca/osiris/data-products.php#OSIRISLevel2DataProducts. SAGE III/ISS data are available at https://asdc.larc.nasa.gov/project/SAGE%20III-ISS/g3bssp_51. OMPS aerosol data are available at https://zenodo.org/record/4029555. ACE data are available through the following sign-up link: https://databace.scisat.ca/l2signup.php. OMI ozone data were obtained from https://acd-ext.gsfc.nasa.gov/anonftp/toms/).
