# Supplementary material for: On the stratospheric chemistry of midlatitude wildfire smoke
Source: Proc Natl Acad Sci U S A. 2022 Mar 1;119(10):e2117325119. doi: 10.1073/pnas.2117325119 (PMC8915979; doi:10.1073/pnas.2117325119)
Supplement: Supplementary File [file pnas.2117325119.sapp.pdf]

PNAS
www.pnas.org

Supplementary Information for
On the Stratospheric Chemistry Of Mid-Latitude Wildfire Smoke

Susan Solomon, Kimberlee Dube, Kane Stone, Pengfei Yu, Doug Kinnison, Owen B. Toon, Susan E. Strahan, Karen H. Rosenlof, Robert Portmann, Sean Davis, William Randel, Peter Bernath, Chris Boone, Charles G. Bardeen, Adam Bourassa, Daniel Zawada, Doug Degenstein

Corresponding author: solos@mit.edu

This PDF file includes:

Figures S1 to S5

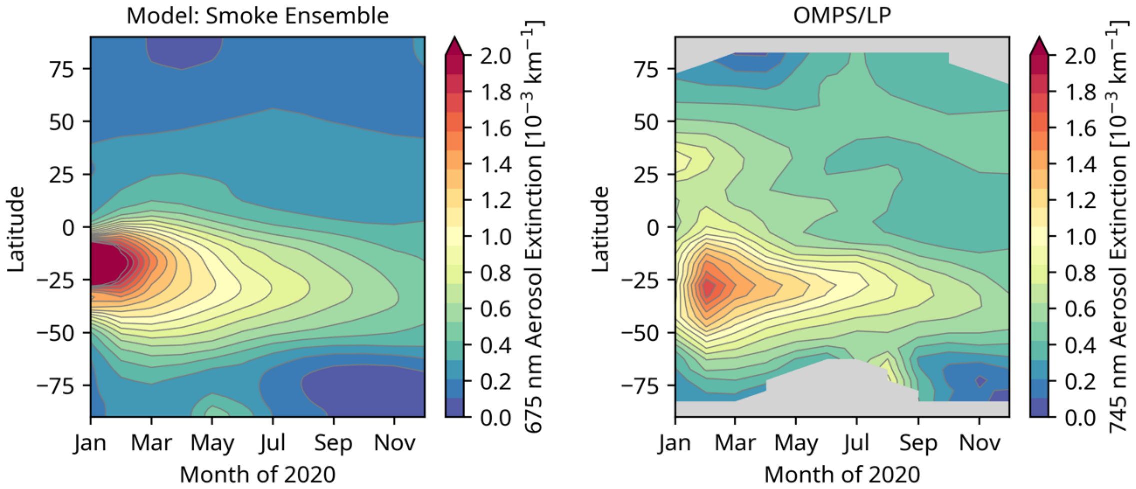

Figure S1. Time series of extinction versus latitude at 18.5 km in 2020 for the model at 675 nm (left) and the OMPS observations at 745 nm).

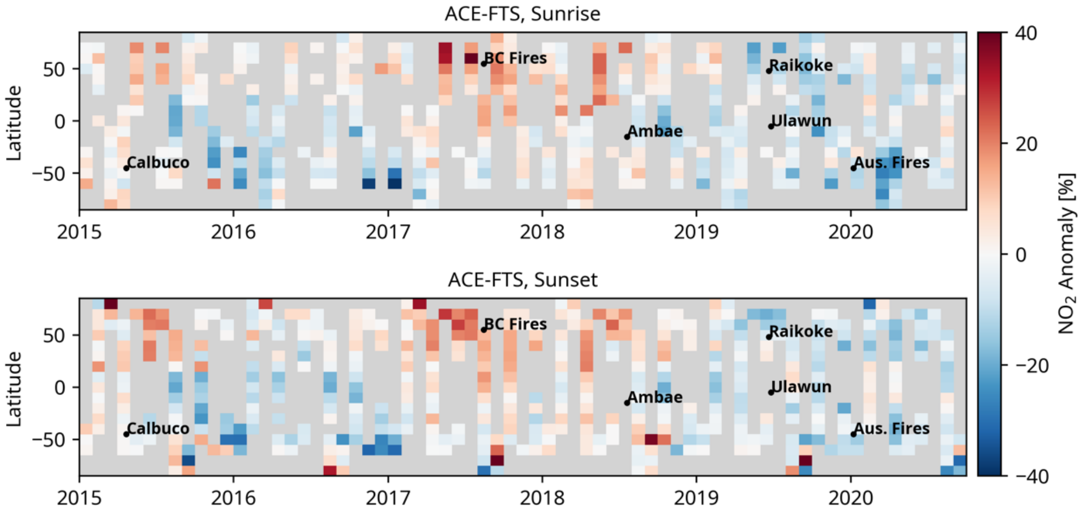

Figure S2. Monthly averaged de-seasonalized NO_2 anomalies at 18.5 km (percent) for ACE sunrise (top) and sunset (bottom), as in Figure 2 for OSIRIS and SAGE III/ISS NO_x.

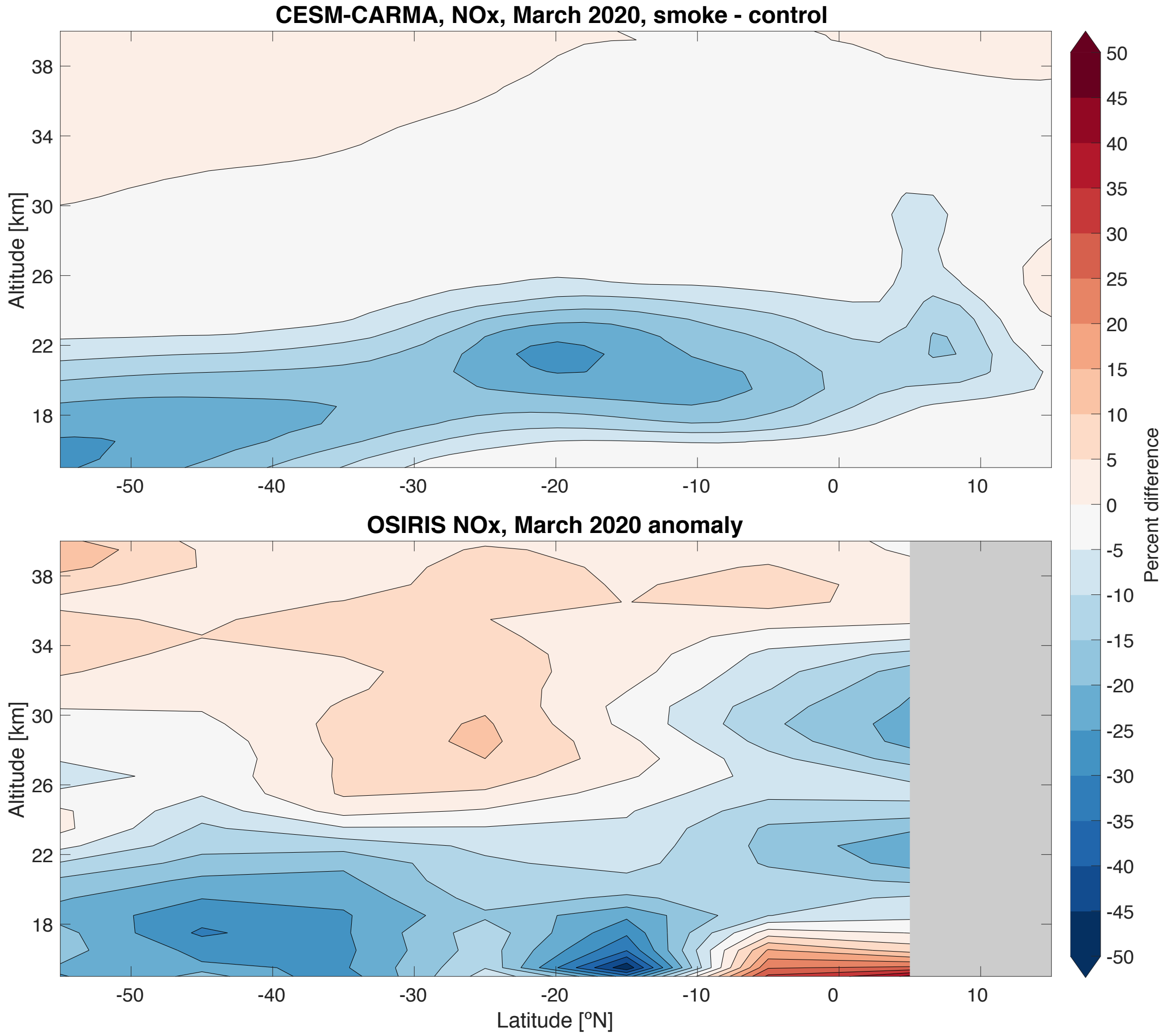

Figure S3. Latitude versus altitude plots of model smoke minus no smoke (top) for March 2020 compared to the observed OSIRIS monthly mean NOx anomaly (bottom). The Ulawun eruption (see Figure 1 of the main text) likely contributed to the low tropical NOx in OSIRIS, and was not included in the model.

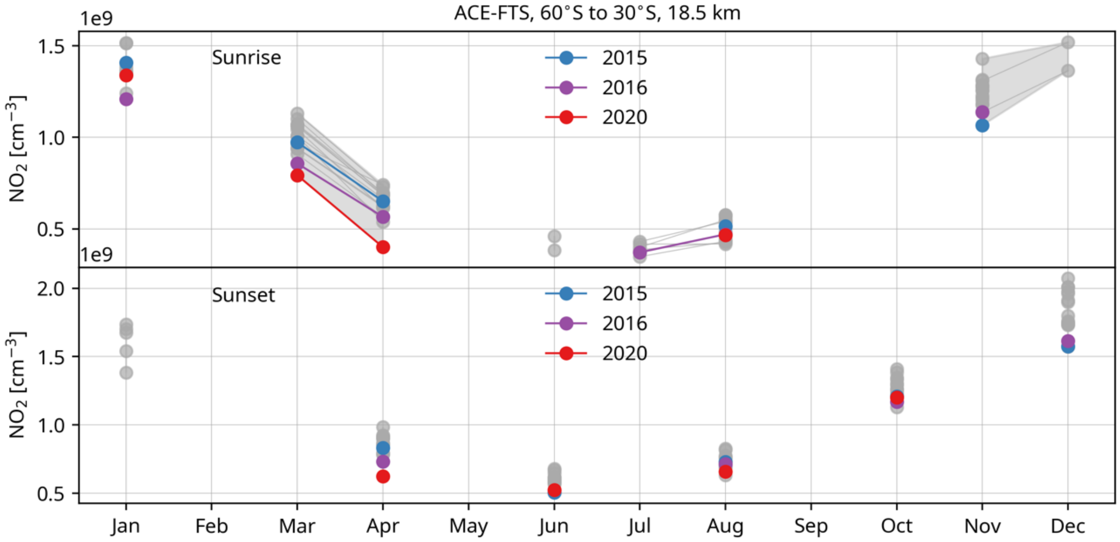

Figure S4. NO_2 concentrations (molecules/cm^3) by month for ACE data over 2004 to 2020 averaged from 30-60°S at 18.5 km, as in Figure 4 of the main text for OSIRIS NO_x. Sunrise data are presented at the top while sunset is at the bottom. 2015, 2016, and 2020 data are indicated by colored points while other years are denoted in gray.

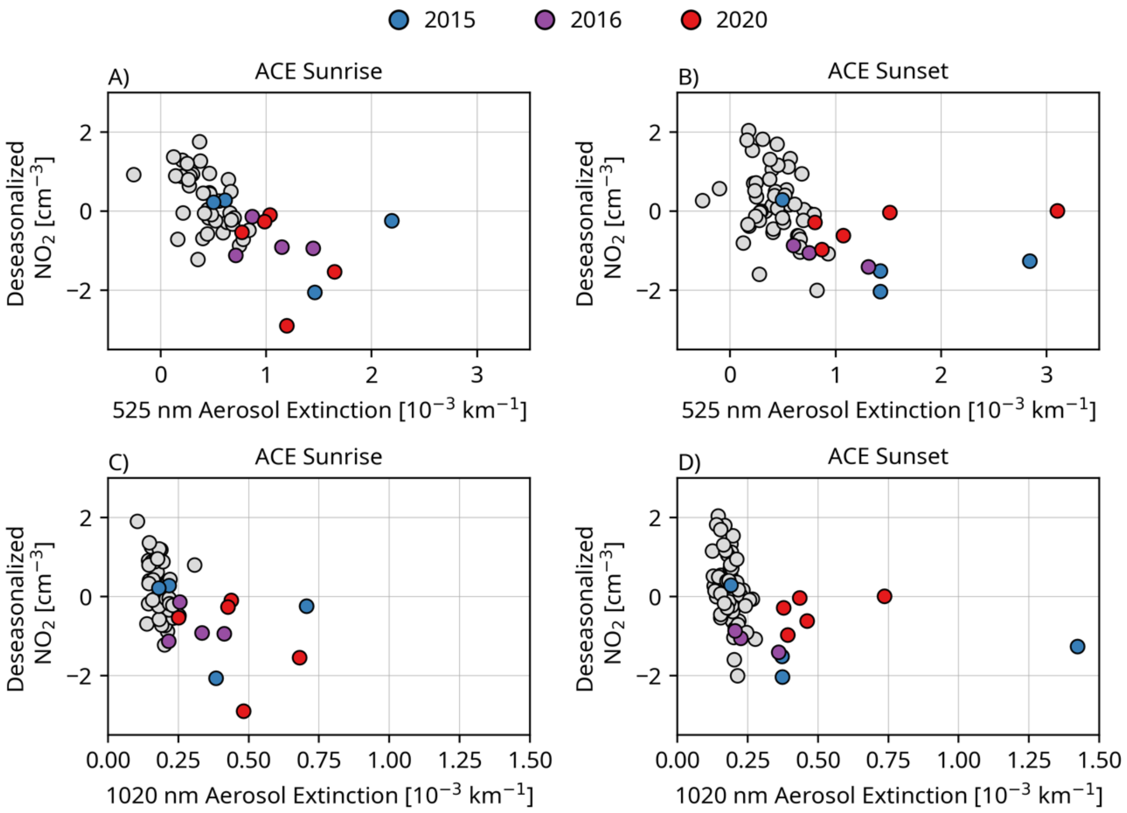

Figure S5. De-seasonalized monthly averaged NO_2 and aerosol extinction anomaly data from ACE for 2020 at 18.5 km from 40-45°S. Sunrise and sunset NO_2 versus 525 nm extinction are shown in panels A and B, while panels C and D present the NO_2 versus 1020 nm extinction. Outliers greater than 4 median absolute deviations from the median were removed from the data. Note that the wavelengths of available ACE extinction data are different from those of OSIRIS and SAGE III/ISS presented in Figure 4 of the main text. 2015, 2016, and 2020 data are indicated by colored points while other years are denoted in gray.
